# Field sizes and the future of farmland biodiversity in European landscapes

**DOI:** 10.1111/conl.12752

**Published:** 2020-10-05

**Authors:** Yann Clough, Stefan Kirchweger, Jochen Kantelhardt

**Affiliations:** ^1^ Faculty of Science Centre for Environmental and Climate Research Lund University Lund Sweden; ^2^ Study Centre for International Analyses (STUDIA) Schlierbach Austria; ^3^ Department of Economics and Social Science, Institute of Agricultural and Forestry Economics University of Natural Resources and Life Sciences Vienna Austria

**Keywords:** agrienvironmental policy, agricultural land consolidation, defragmentation, ecological economics, farmland biodiversity, field edges, land fragmentation, landscape configuration, mechanization, plot size

## Abstract

Lower diversity of plant and animal farmland species are usually reported where cropland has been aggregated into larger fields, which raises prospects of curbing declines in European farmland biodiversity and associated ecosystem services by halting trends to field size increases associated to agricultural intensification, without having to set aside arable land for conservation. Here, we consider the factors underlying trade‐offs between farmer income and biodiversity as mediated by field size at local and landscape scales, and how these trade‐offs may be overcome. Field sizes are still increasing, facilitated by increasing farm sizes and land consolidation. Decreases in working time and fuel expenses when fields are larger, uptake of larger machinery and subsidies favoring larger farms provide incentives to manage land in larger units, putting farmland biodiversity further at risk. Yet, field size‐mediated ecological–economic trade‐offs are largely ignored in policy and research. We recommend internalizing the ecological effects of changes in landscape‐scale field size into land consolidation scheme design, ensuring that EU Common Agricultural Policy post‐2020 rewards farmers that maintain and recreate fine‐grained landscapes where these are essential for farmland biodiversity targets, and reducing economic–ecological trade‐offs by stimulating agricultural research and innovation for economically efficient yet biodiversity‐friendly farming in fine‐grained landscapes.

## 1 INTRODUCTION

Agricultural landscapes have been subjected to profound changes in historical and contemporary times, including spatial separation of animal husbandry and arable crops, reduced diversity of arable crops, and disappearance of seminatural habitats (Benton, Vickery, & Wilson, 2003). These changes have led to significant declines in the diversity, abundance and biomass of many noncrop organisms (e.g., Brooks et al., 2012; Gregory, Skorpilova, Vorisek, & Butler, 2019; Hallmann et al., 2017; Seibold et al., 2019).

Besides altered landscape composition, the configuration of the landscape has changed as field size increased, accompanying mechanization, the separation of animal and arable farming, collectivization, and land consolidation programs (Skaloš, Molnárová, & Kottová, 2012; Tryjanowski et al., 2011). This often caused a reduction in field margins, including grass strips, hedgerows and ditches, which are important refuges for biodiversity (Baude et al., 2016; Marshall & Moonen, 2002; Sklenicka et al., 2009). Since arable landscapes are often mosaics of fields with different crops, larger field sizes result in coarse‐grained landscapes, with lower diversity of crop types, field edges, and noncrop habitats (Baguette & Van Dyck, 2007; Turner, O'Neill, Gardner, & Milne, 1989).

**FIGURE 1:**
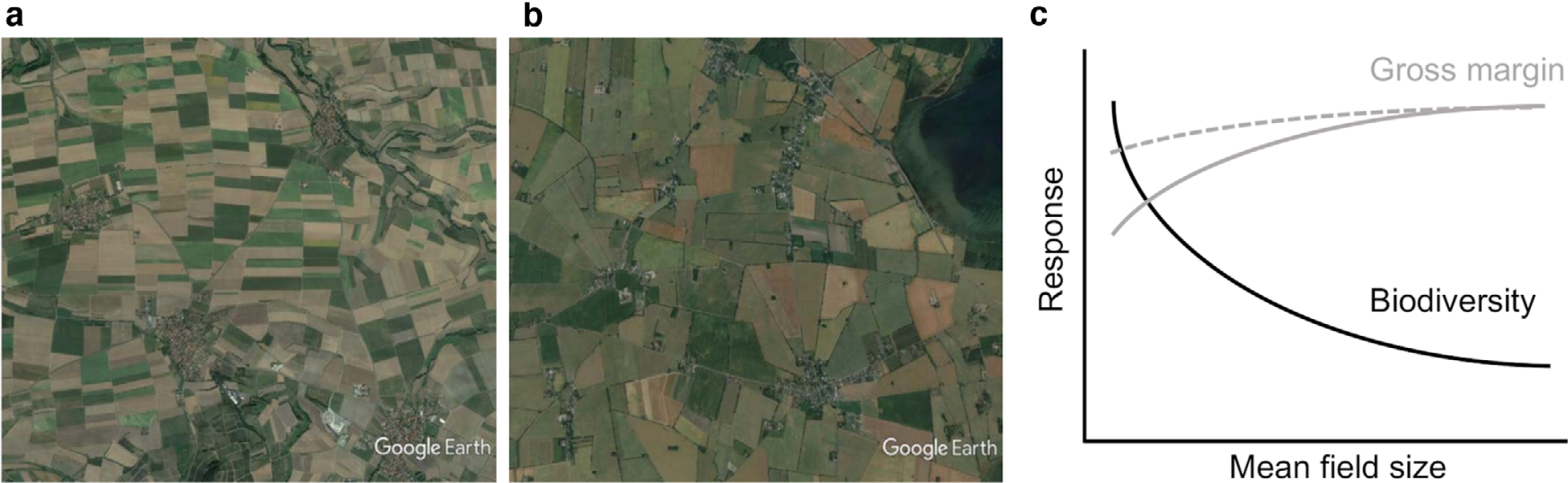
Landscapes in Lower Franconia, Germany, with mean field size around 2 ha (a), landscape in Scania, southern Sweden, with mean field size around 12 ha (b), and expected ecological–economic trade‐off mediated by field size (c), and effect of lessening the ecological–economic trade‐offs (gray to gray stippled) explored in the text. The exact relationship will vary between landscapes and regions given both the socio‐economic context and the biodiversity targets

Very recently several comprehensive studies from Europe and North America demonstrated that the mean size of agricultural fields in a landscape (e.g., Figure 1a, b) is a major driver of diversity and abundance of farmland biodiversity taxa including plants, arthropods, and vertebrates (Martin et al., 2019; Sirami et al., 2019, see also below for further references).

A conclusion of these studies is that small field sizes are of utter importance to halt and maybe even reverse the decline in biodiversity in landscapes dominated by annual crops, which cover 19% of the terrestrial land area (Václavík, Lautenbach, Kuemmerle, & Seppelt, 2013). It has also been suggested that reducing field sizes is feasible without reducing food production, something which strategies that involve either setting aside land for conservation or reducing intensity (e.g., organic farming) often do not achieve (but see Pywell et al., 2015). However, economic profits, not crop yields, drive farmer decisions. While there is evidence for ecological–economic trade‐offs between farmer income‐ and biodiversity‐related indicators incurred by reducing field size for increasing biodiversity (Rodriguez & Wiegand, 2009), the existing knowledge on drivers and consequences of changes in agricultural landscape grain is dispersed across scientific disciplines (agricultural economics, landscape ecology, conservation biology, geography).

Here, we jointly consider evidence for (1) the links between field size and biodiversity, including the underlying processes, (2) the historical background of differences in field sizes, (3) the farm economic effects of field size, including through ecosystem services to the crops, (4) trends in agricultural practices and agricultural policy and their likely impacts on field size, and (5) provide recommendations for conserving and restoring fine‐grained agricultural landscapes to support biodiversity conservation in agricultural landscapes, amongst others in the current reform of the Common Agricultural Policy of the EU. While our focus is largely on European arable‐dominated landscapes with mechanized agriculture, many of the reviewed processes may apply more generally, including to parts of North America (Fahrig et al., 2015) and rice growing landscapes in East and South East Asia (Dominik et al., 2018; Katayama et al., 2015).

### 1.1 Why is farmland biodiversity higher in landscapes with smaller fields?

The evidence for higher diversity and abundance of organisms in landscapes where the agricultural area is subdivided into smaller fields stems from studies using existing differences in field size between landscapes. These include both contrasts across the former political border dividing Europe into East and West (Batáry et al., 2017; Šálek et al., 2018) and the general variation in mean field size within regions in Europe (Concepción et al., 2020; Hass et al., 2018; Konvicka, Benes, & Polakova, 2016; Martin et al., 2019; Sirami et al., 2019; Skórka, Lenda, Moroń, & Tryjanowski, 2013; Zellweger‐Fischer et al., 2018) and North America (Fahrig et al., 2015; Monck‐Whipp, Martin, Francis, & Fahrig, 2018), covering multiple taxa and controlling for field‐scale management intensity. The effect sizes are significant, since moving from a field size of 1–6 hectares has a similar negative effect on farmland biodiversity as the difference observed when moving from 35% to 0% seminatural habitat cover in the surrounding landscape. Effect sizes were also found to match the difference in biodiversity between organic and conventionally managed fields (Batáry et al., 2017; Belfrage, Björklund, & Salomonsson, 2005). The rate of decrease in species richness with increasing mean field size is larger in the range between 0.25 and 3 ha than in the range between 3 and 12 ha, consistently across different regions and taxonomic groups (Sirami et al., 2019). Increases in mean field size affect biodiversity through several mechanisms (Dunning, Danielson, & Pulliam, 1992). First, it leads to a decrease in the density of field edges. These can be either field‐field ecotones without any permanent vegetation, or permanent field edges including grassy strips, hedges, walls, ditches, or any combinations thereof. Permanent field edges, especially, act as refuges for many species, providing nesting sites and food resources. Crop–crop borders, even without any permanent vegetation, can facilitate movement of flying insects across cropland (Hass et al., 2018). Second, increasing field sizes decreases the diversity of crops at finer spatial scales (Fahrig et al., 2011), which can decrease the abundance of mobile species that move between different crops, for example, where they find complementary resources (Smith et al., 2014). Third, through a sampling effect, increasing field sizes can cause certain crops to be absent from the landscape in a given year, thereby decreasing crop diversity even at larger spatial scales. Both mean field size/density of field margins and crop diversity were found important in studies whose design allowed separation between their respective effects, with effects of mean field size being somewhat stronger and more consistent across species groups (Sirami et al., 2019; Skórka et al., 2013). Landscape‐scale field sizes affect both common species and rare species. Field margins and the edges of arable fields have been shown to be important for rare and red‐listed species among plant, bryophytes, and birds (Gabriel, Roschewitz, Tscharntke, & Thies, 2006; Wuczyński, Dajdok, Wierzcholska, & Kujawa, 2014). Both theories that consider the landscape to be subdivided into habitat or nonhospitable matrix (species‐area relationships, meta‐population theory), as well as those that focus on the use of multiple habitats (source‐sink processes and habitat complementation/supplementation) are helpful in framing the effects of field sizes on biodiversity. The relative relevance of these two theories to a particular context depends on the degree of habitat specialization and mobility in the species or ecological functional groups being considered (Dunning et al., 1992; Smith et al., 2014). Habitat generalists, such as many farmland wild bee species, may be dependent on finding a diversity of resources, and asynchronous disturbance, within their foraging ranges (Hass et al., 2018) while specialists (e.g., grassland specialists such as fritillary butterflies; Cozzi, Müller, & Krauss, 2008) may be more sensitive to the availability of habitat in the landscape irrespective of the configuration of the arable landscape. Large arable fields can be preferred by certain wintering and migrating birds (Gillings, Fuller, & Sutherland, 2007; Rosin et al., 2012), as well as open grassland birds, which can be particularly important conservation targets (e.g., puszta and Iberic cereal plains; Báldi & Batáry, 2011; Concepción & Díaz, 2019). The preference of individual bird groups for large over small fields is variable between regions (Concepción & Díaz, 2019), even within species (e.g., litte bustard; Báldi & Batáry, 2011; Silva, Palmeirim, & Moreira, 2010). Thus, while overall evidence for higher biodiversity levels in landscapes where smaller fields have not been aggregated into larger units is overwhelming and justify greater attention in conservation, there are important farmland species that thrive in large fields when adequately managed, and the optimal field size may differ between regions and conservation targets. Thus, while overall evidence for higher biodiversity levels in landscapes where smaller fields have not been aggregated into larger units is overwhelming and justify greater attention in conservation, there are important farmland species that thrive in large fields when adequately managed, and the optimal field size may differ between regions and conservation targets.

### 1.2 The historical background for differences in field sizes

Field sizes differ by one to two orders of magnitude in Europe (Herzog et al., 2006; Figure 1a,b) and at least four orders of magnitude globally (Kamp et al., 2012). Landscape‐wide patterns of field size are linked to patterns of land ownership and to land management. Fragmentation of land ownership and land‐use occurs through inheritance and use of land as a means of payment. It is accompanied by counteracting defragmentation processes to ensure practicability of farming, and both are near‐continuous processes that have shaped the spatial grain of agricultural landscapes since at least medieval times (Vitikainen, 2004). Land reforms resulted in rapid increases in mean field sizes in different parts of Europe in the 19th century (Skaloš et al., 2012). Agricultural intensification from the 1930s–1940s onward, and in particular the access to modern machinery, have led to further increases in mean field size (Ihse, 1995; Robinson & Sutherland, 2002; Skaloš et al., 2012). In those countries of the former socialist bloc where collectivization was imposed, arable fields were consolidated, leading to very large field sizes (Hartvigsen, 2014). In some cases, but not all (Hartvigsen, 2014), restitution and distribution processes after the lifting of the Iron Curtain have led to subsequent decreases in field size (Skaloš et al., 2012; White & Roy, 2015). The resulting pattern is one of high regional variation in field sizes that is not only explained by topographical conditions or farm size, and where the current field size distribution may not always be perceived optimal for modern farming.

### 1.3 Field sizes, crop productivity, and farm economics

Smaller arable fields may be associated with increased inputs per area in terms of both working time and other inputs, decreased yields, increased opportunity costs and decreased value of farmland (Gonzalez, Marey, & Alvarez, 2007; Latruffe & Piet, 2014; Figure 1c), but also with increased biodiversity‐mediated ecosystem services provided to crops (Martin et al., 2019).

In mechanized agriculture, field operations such as sowing, tillage, input application and harvesting involve starting at the edge of the field and accelerating to the speed of operation, driving along the field, usually the long side, then slowing down and turning around the machinery at the end of the field. A field size of 1 ha, when compared to a field size of 5 ha, can imply an increase of 30–90% in working time per unit area spent on machine operations, depending on the work width (e.g., width of the spray boom or tillage implement) and the speed of operation (Nilsson, Rosenqvist, & Bernesson, 2015). Since smaller field sizes imply longer operations in the field (Nilsson et al., 2015), the amount of fuel consumed will also be higher with small fields than large fields (e.g., Kapfer, 2007; Latruffe & Piet, 2014). The area at the end of the field on which the machinery turns is the headland, sometimes called turning headland (in which case the field borders are nonturning headlands) (Sparkes, Jaggard, Ramsden, & Scott, 1998). This area is more heavily trafficked, and there is a higher risk for overlapping application of inputs. Smaller fields lead to an increasing share of headlands per unit area, and might therefore lead to higher input use (fertilizer, pesticides). Overall, large increases in variable costs with smaller field sizes, especially when below ∼2 ha, apply to all arable crops but also permanent (mown) grassland (Kapfer, 2007), and have been observed not only for arable but also for dairy farming (Hansson, 2007).

Smaller fields can be associated with lower yields (Latruffe & Piet, 2014), but the exact causes for these patterns have not been well investigated. The headlands involve repeated passage of machinery, which can cause soil compaction and damage to the crop itself. The decreases in yields reported in different studies for headlands and field edges are summarized in Table S1. As for the effects on variable costs, field size matters most at smaller size ranges, where the headlands contribute a significant area of the field. In regions where the smallest fields are already large (e.g., at ranges 30–300 hectares) yields do not correlate with field size (Robertson, Lyle, & Bowden, 2008).

Given its potential effects on yields, and especially on input costs, field area is a factor that can affect pricing by contractors and the calculation of value of farmland (Oksanen, 2013). Extreme land fragmentation can significantly depress land prices on the sales and/or rental market (Sklenicka, Molnarova, Pixova, & Salek, 2013).

While negative effects of field size on field‐ and farm‐level economic performance seem to predominate, smaller field sizes also have economic benefits (King & Burton, 1982). Heterogeneity across the farm and uncertainty regarding growing conditions is an incentive to avoid growing a crop in a single field to reduce risk of crop failure. This is most obvious in very heterogeneous areas where farms cover large natural (e.g., altitudinal) gradients, but is also valid when heterogeneity is less extreme. Very large fields may have their own logistical challenges in terms of field operations, and can make it more difficult to adjust the management to the field conditions. However, these limitations can be partially overcome using sensor techniques and satellite controlled farm machinery, which allow precision farming and site‐specific management.

Higher biodiversity in landscapes with small fields supports ecosystem services in crops. Wild pollinators such as solitary bees and bumblebees nest mainly outside of arable fields, and are limited in the distance they are able to forage into a flowering crop. Accordingly, the contribution of pollinators to yields is greater in landscapes dominated by small rather than large fields (Garibaldi et al., 2016). A recent synthesis found that in landscapes with smaller fields, 70% of pollinator and 44% of natural enemy species—in particular flying and ground dwelling predators that do not overwinter in the crop—reached highest abundances and pollination and pest control improved 1.7‐ and 1.4‐fold, respectively (Martin et al., 2019). Where landscapes were arable‐dominated, smaller fields in the landscape led to higher yields (Martin et al., 2019), though this, when integrated over space, may be somewhat offset by lower yields at the field borders.

While differences in variable costs between large and small fields apply to all crops and are very visible to the farmer on a day‐to‐day basis, benefits of strengthened crop pollination and natural pest control, and their effects on yield, are likely less visible. These rely on ecological interactions that are difficult to observe, with pest pressure and crop pollination requirements being variable between seasons, crops, and varieties. Pesticide use and the reliance on managed pollinators such as placing honeybee hives near large oilseed rape fields further contribute to hiding the beneficial effects of smaller field sizes. We hypothesize that a choice to implement sustainable intensification with an increased reliance on ecosystem services rather than inputs (Bommarco, Kleijn, & Potts, 2013), would lead to smaller field sizes being economically more advantageous than is the case under the current paradigm, where larger fields are economically more attractive for farmers.

### 1.4 Current and future trends in agricultural practices and agricultural policy and their likely impacts on field size

#### 1.4.1 Defragmentation

Small field sizes, large number of fields per farm area, small farm area and spatially dispersed fields are interlinked dimensions of land fragmentation that are considered to hamper economically efficient, or “economically rational” farming, given current farm structure and technology (King & Burton, 1982; Van Dijk, 2003, see also below). The main processes that can reverse all or some facets of land fragmentation are defragmentation of ownership by land consolidation projects (often with financial support from the government; Noleppa et al., 2008; Vitikainen, 2004) or land sales, and defragmentation of land‐use by rental market. Local or regional land consolidation, or reallocation, is an ongoing process across much of the old world's farmland, including Northern Europe, Southern Europe and Asia (Crecente, Alvarez, & Fra, 2002; Janus & Markuszewska, 2017; Jiang et al., 2017; Johansen et al., 2018). The principal aims of land consolidation projects may encompass the maintenance of the viability of farming, the facilitation of urbanization or infrastructure projects, or the creation of protected areas or other elements of green infrastructure (Vitikainen, 2004). They can result in significant changes to the landscape grain size, but are costly and often take a decade of planning. The land markets where plots can be sold, leased, subcontracted, or swapped also offer opportunities for farmers to defragment land‐use. In principle a quick and cost‐effective means for economically efficient distribution of the land, land markets are imperfect (Sklenicka, Janovska, Salek, Vlasak, & Molnarova, 2014), and primarily used to increase farm size rather than defragment the land.

Field sizes are thus dynamic and affected by multiple bottom‐up and top‐down processes that operate at multiple scales. These processes are ongoing, but most are slow and play out in the long‐term. Even where environmental assessments inform the process, such as in many land consolidation/reallocation projects, the effects of changes in farmland grain size on biodiversity and ecosystem services are rarely considered.

#### 1.4.2 Structural change

The number of farms has been constantly decreasing in Europe, with the remaining farms increasing in size as the land enters the rental market and is rented by other farms (e.g., EUROSTAT 2018, data 2005–2013). There is evidence for systematic increases in field size as farm size increases over time (Levin, 2006). This may cause rapid land‐use change in some regions. In Poland, which has a large range of field sizes and a substantial number of small farms and fields with high biodiversity value, increase in land concentration and in the number of large farms, increase in mechanization and decrease in farm labor have accelerated , when comparing the period 2000–2011 with 1990–2000 (Chmielinski & Karwat‐Wozniak, 2015). Field size increases follow increases in farm size, but to which degree, and at what speed, may vary. If the pressure on land is high, increases in farm size may need to be achieved through renting land as it comes onto the market, even if it is in the form of smaller fields at a distance from the farm center, so in the short‐term field size may not automatically increase with farm size (Barbottin, Bouty, & Martin, 2018; Forbord, Bjørkhaug, & Burton, 2014).

Where farm and field sizes are too low to allow economically viable farming, and in the absence of economic compensation or alternative sources of income that contribute to maintain farming, abandonment of farming has similarly deleterious effects on farmland biodiversity (Queiroz, Beilin, Folke, & Lindborg, 2014), even though at least temporary positive effects have also been reported (Skórka & Lenda, 2009). Another potential danger in regions where land fragmentation is not economically profitable is the renting out of plots by smallholders to larger operations. For example, in Slovakia the renting‐out of small plots too small to farm to corporate farms that aggregate the small plots into large field units has been documented (Sklenicka et al., 2014). This can lead to dramatic and comparatively rapid changes in scale and intensity of agricultural practices.

#### 1.4.3 Changes in subsidies to the farming sector

Despite efforts to decouple agricultural production from governmental payments, the structure of the farming sector and the land‐use in agricultural landscapes, including field size, is still strongly influenced by the existence and modalities of subsidies. In the European Union (EU), subsidies under the Common Agricultural Policy (CAP) account for a large share of farmer income. CAP effects on field size have not been assessed explicitly. However, predictions of what would happen if CAP payments were removed altogether include widespread abandonment in less productive areas that are associated with smaller field sizes, and decreases in farm numbers and increases in farm sizes in more productive areas (Johansson et al., 2017; Kapfer, Ziesel, & Kantelhardt, 2015; Piet, Latruffe, Le Mouël, & Desjeux, 2011). The latter could entail further enlargements of fields where this is currently constrained by ownership fragmentation. Furthermore, under the pillar II of the CAP, additional payments are handed out to farmers in areas identified as “least favorable” for farming or “under natural constraints” (ANC), which are associated with low productivity (Jones et al., 2012). This may benefit farmers in landscapes with smaller fields either indirectly, since areas with lower productivity tend to have smaller fields, or directly, whenever the states hand out extra ANC payments for managing fields below a certain size (e.g., in Austria and Bavaria). Such additional payments also occur in the framework of certain agrienvironmental schemes (e.g., KULAP in Bavaria) to prevent abandonment of smaller parcels. However, while the existence of CAP payments to farmers may cause farms to be smaller than if the payments were absent, the modalities of the CAP have also been criticized for disadvantaging small farms (van der Ploeg, Franco, & Borras, 2015). Subsidies to farmers are based on the area farmed, which makes increasing farm size an attractive way to increase total amount of subsidy payments and thereby income. Furthermore, basic payments in 2006–2013 were only eligible for fields with a minimum parcel size of 0.1 hectares (COMMISSION REGULATION (EC) No 263/2006), a limit that was set higher by some EU member states, with, for example, 0.3 ha in Romania (Mikulcak, Newig, Milcu, Hartel, & Fischer, 2013). In the current CAP period, eligibility is restricted by farm size, ranging from 0.3 to 5 ha depending on the member state (DG AGRI Direct payments 2015–2020 Decisions taken by Member States: State of play as at June 2016, Information Note). This likely continues to bias aid away from the smallest field sizes.

### 1.5 Steps toward conserving and restoring fine‐grained agricultural landscapes

**FIGURE 2:**
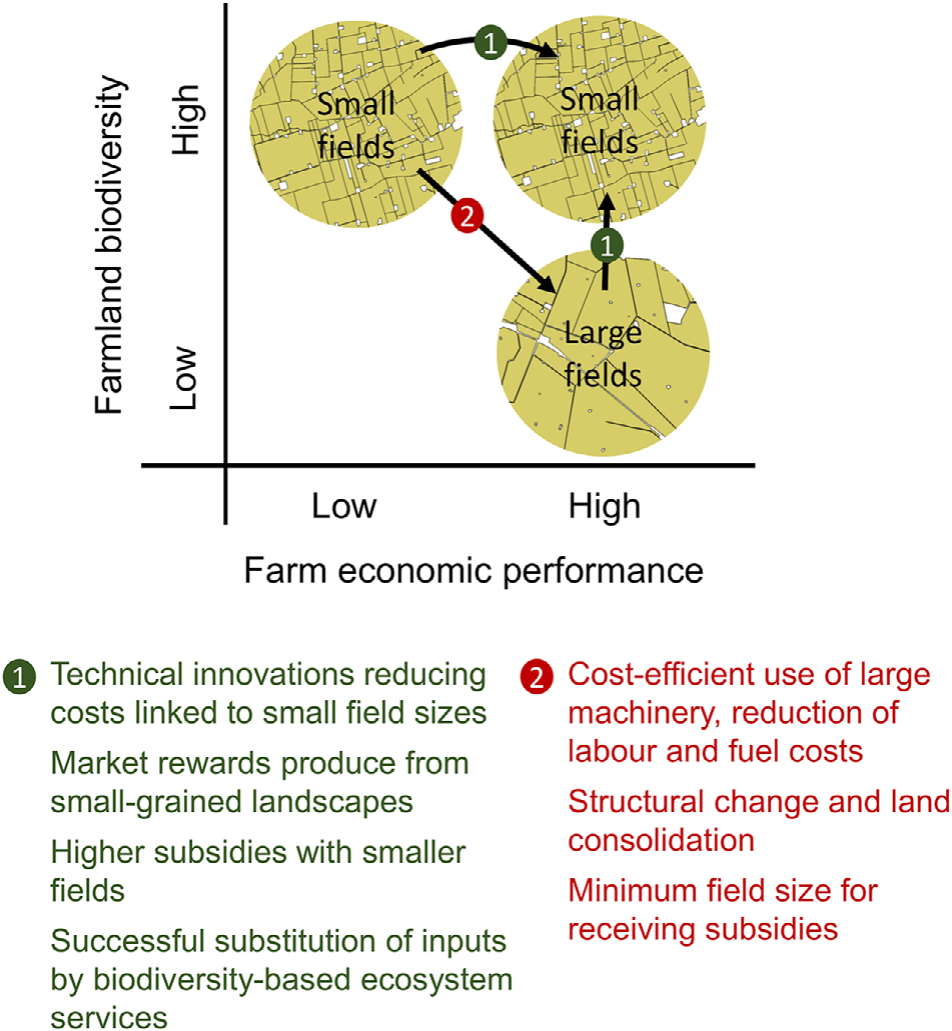
Factors identified in the review that can contribute to transitions to and from fine‐grained to coarse‐grained agricultural landscapes, and from lower to higher farm‐economic viability of fine‐grained landscapes

The ecological–economic trade‐offs associated with increases in field sizes are significant. To curb future biodiversity loss due to increases in field sizes, and based on the available evidence, several measures can be recommended (summarized in Figure 2).

#### 1.5.1 Rewarding farmers for biodiversity benefits of landscapes with small fields

The economic–ecological trade‐offs of decreasing field sizes show that targeted support is needed for farmers to maintain active agriculture with small field sizes. Capitalizing on small‐scale agricultural landscapes may be achieved in different ways in the ongoing reform of the CAP. Abolishing or reducing the minimum parcel size for eligibility for basic payments under the CAP would reduce the contradiction between instruments for supporting biodiversity in agricultural landscapes under the CAP, widely criticized for being ineffective (Pe'er et al., 2014), and excluding farms with very small fields that are particularly beneficial for biodiversity from the most basic level of subsidies. Furthermore, under the current logic of payments for actions that support biodiversity, EU member states’ CAP strategic plans (CSPs) could reward farmers that maintain smaller fields in regions where this is effective in supporting farmland biodiversity. Examples would be point‐based ecoschemes (Lampkin et al., 2020), where smaller mean field size could contribute toward increasing payments. Landscape‐scale mean field size can also be more widely included in the definition of areas under natural constraints (ANC, formerly “least‐favored areas”). We emphasize that field size targets cannot be defined top‐down at the EU or national level, nor do we see the solution in region‐wide thresholds of field size. While field size can be a very effective lever for biodiversity conservation in many landscapes, the optimal measure to improve farmland biodiversity—and the optimal landscape‐scale field size—will be dependent on the biodiversity target and the land use context (management intensity, share of seminatural habitat) in a given region or landscape (Concepción & Díaz, 2019). In the new CAP post2020, CSPs should take this heterogeneity into account by adjusting recommended measures, and payment levels, to local conditions (Báldi & Batáry, 2011; Concepción & Díaz, 2019). It has been suggested that rather than relying entirely on payments to individual farmers, fostering result‐based collaborative and community‐led approaches may be more promising to maintain fine‐grained agricultural landscapes in the long term (Fischer, Hartel, & Kuemmerle, 2012; Herzon et al., 2018; Wästfelt, Saltzman, Berg, & Dahlberg, 2012). This may be compatible with market‐based approaches, for example through added‐value of certified and labeled *terroir* products that often stem from traditional farming associated with small fields (Angelstam, Boresjö‐Bronge, Mikusiński, Sporrong, & Wästfelt, 2003).

#### 1.5.2 Break the economic–ecological trade‐off: reduce costs associated with small fields

The potential for using cutting‐edge technology to increase the economic viability of fine‐grained landscapes and thus conserve farmland biodiversity is nearly unexplored. We showed above that the ecological–economic trade‐offs in maintaining small fields are largely due to the difficulty in effectively using modern machinery in small fields (Gonzalez et al., 2007; Rodriguez & Wiegand, 2009). Adapting field shape with by increasing perimeter/area ratio and minimizing headlands, resulting in longer, narrow fields may improve economic (Gonzalez, Alvarez, & Crecente, 2004) as well as ecological performance (see Fernández, Acosta, Abellá, López, & Díaz, 2002 for a theoretical framework integrating multiple edge effects relative to patch size and shape). Interestingly, there is increasing research into adapting technology for small fields (Aravind, Raja, & Pérez Ruiz, 2017; Duckett, Pearson, Blackmore, & Grieve, 2018), driven by the recognition that globally, small farms and fields are still the norm, and that the potential for securing market shares of agricultural machinery by further increasing machine size is limited. The use of fleets of small fossil‐free autonomous vehicles may not only make it possible to farm smaller fields effectively, but also both reduce the environmental costs in terms of fossil fuel use and help maintain biodiversity levels with no or lower levels of subsidies though agrienvironmental support. Furthermore, technology might be used to create artificial field elements, either perennial, for instance where productivity is low very locally, or annual. For example, satellite controlled farm machinery could automatically leave out the cultivation of small areas within fields in order to create, to maintain and to care for small, annual biotopes in large, fairly homogeneously cultivated fields. Alternatively, various crops could be planted at very small scale next to each other, leading to higher field edge densities is currently the rule. Fields need not even be more or less rectangular, but could for instance be adapted to the configuration of underlying soil types.

#### 1.5.3 Integrate biodiversity effects of increasing field sizes in the environmental impact assessment of land consolidation schemes

Land consolidation schemes have increasingly taken into consideration environmental aspects, and loss of field edges is often compensated using some form of land sparing approach, by the (re)creation of larger blocks of green space and or seminatural habitats, for purposes that may include nature conservation and recreation, or other ecosystem services (Benthem, 1969; Kapfer, 2007). The dichotomy between farmland (the “matrix”) and unfarmed land (the “patch” or “patches” of noncrop habitat) mean that positive effects on farmland biodiversity and associated ecosystem services of having fragmented undisturbed areas, such as a tight net of field edges, and small‐grained landscapes are often ignored (Andrieu, Vialatte, & Sirami, 2015). The integration of recent scientific findings on the importance of small field sizes for farmland biodiversity into the planning of land consolidation schemes would make decision‐makers consider options that maintain a fine‐grained landscape while reducing other dimensions of land fragmentation.

#### 1.5.4 Using field size as an indicator of biodiversity friendliness at farm to regional scale

The environmental consequences of farm size are a recurring debate in discussions on agricultural policy, since farm size has always been an easily available indicator from farm to regional level. Here, we have shown that substantial evidence points toward field size being a major driver of farmland biodiversity, alongside cover of seminatural habitat and intensity of the land‐use within the fields. Field size is related to farm size, but not to the extent that these are substitutable (Levin, 2006), and while field size has previously been more difficult to quantify than farm size, this is no longer the case. In the EU, field size information is available based on polygon data from GIS systems linked to subsidy administration and control systems, the IACS‐LPIS (Sagris, Wojda, Milenov, & Devos, 2013), thus making this available to administrators. Less precise but useful for monitoring trends, methods to assess patterns of field size using field texture have been developed and applied in mapping exercises (Kuemmerle, Hostert, St‐Louis, & Radeloff, 2009; Weissteiner, García‐Feced, & Paracchini, 2016). Sample based categorical field size data is available in Europe through the point‐based LUCAS system. We recommend this information be used to complement existing habitat‐based indirect biodiversity indicators at farm to regional scale (Herzog et al., 2017). At the farm scale, field size could be taken up as an additional indicator in app‐based tools used for on‐farm self‐monitoring by farmers (e.g., Cool Farm Tool https://coolfarmtool.org/coolfarmtool/biodiversity/). The same applies to monitoring and policy assessment, which would benefit from field size being integrated with existing indicators. For example, in the EU, High Nature Value (HNV) farming is a concept well established with policy makers and conservationists to describe the farming systems in Europe of greatest biodiversity value, but hampered by the heterogeneity in indicator availability between members states (Strohbach, Kohler, Dauber, & Klimek, 2015). Here, we suggest taking up field size as a widely available and reliable indicator that can contribute toward identifying potential HNV areas.

## 2 CONCLUSIONS

Recent evidence shows that the increase in field size is an important, but long‐overlooked driver of biodiversity loss in European farmland. The remaining fine‐grained agricultural landscapes are biodiversity strongholds, but field sizes are still increasing due to either abandonment of small fields or consolidation into larger ones. This is a challenge that needs to be addressed, both in order to curb and reverse farmland biodiversity loss, and because smaller field sizes support crop pollination and natural control of pests, and may thus be key in making agriculture sustainable. We suggest three ways forward. First, to further develop the Common Agricultural Policy of the EU in a way such that farmers or farming communities are better financially supported in maintaining small‐grained landscape and supporting market rewards for producing from fine‐grained landscapes will help to increase the profitability of cultivating small plots and improve the effectiveness of current agrienvironmental policy. In view of the great diversity of land management and site conditions for agriculture in Europe, it is important to give regions sufficient scope for designing appropriate measures to foster biodiversity in a subsidiary manner (Díaz & Concepción, 2016). Second, considering the ecological effects of increasing field size in land consolidation schemes could help maintain fine‐grained farmland while addressing other dimensions of land fragmentation, such as ownership fragmentation. Finally, a largely unnoticed, but very promising potential lies in reducing the economic–ecological trade‐off associated with small field sizes. Research and innovation in agricultural machinery and management toward systems that work efficiently with small fields may be key to harness the benefits of fine‐grained landscapes as a part of economically viable sustainable intensification strategies, but research at the interface of such new technologies, farmland biodiversity conservation, and ecosystem service research is almost inexistent.

## AUTHOR CONTRIBUTIONS

YC led the writing, with contributions by SK and JK. All authors contributed to the revisions.

## ETHICS STATEMENT

Does not apply (Review article).

## DATA ACCESSIBILITY STATEMENT

This review relies on published scientific publications and reports. If encountering accessibility issues, please contact the corresponding author of the present review, or the authors of the original paper or report.

## CONFLICT OF INTEREST

The authors report no conflict of interest.

## Supporting information

Supplementary Material
