# Supplementary material for: Field sizes and the future of farmland biodiversity in European landscapes
Source: Conserv Lett. 2020 Oct 5;13(6):e12752. doi: 10.1111/conl.12752 (PMC7816254; doi:10.1111/conl.12752)
Supplement: Supplementary file 1 — Supplementary Material [file CONL-13-e12752-s001.docx]

**Table S1**. Yield depressions at field edges, as reported in the literature. Other data, original source not accessed but cited in ([Lebert, Brunotte, & Sommer, 2003](#_ENREF_8)), 9-17% in winterwheat ([J Brunotte, Weißbach, Rogasik, Isensee, & Sommer, 2000](#_ENREF_3)). 18% crop loss in cereal field in 6m wide edges ([Boatman & Sotherton, 1988](#_ENREF_1)) as cited in ([De Snoo, 1994b](#_ENREF_5)).

| Study | Crop | Type of edge | Width | Yield reduction |
| --- | --- | --- | --- | --- |
| ([De Snoo, 1994a](#_ENREF_4)) | Potato  Sugar beet  Winter wheat | Non-turning  Non-turning  Non-turning | 3m  3m  3m | 10-12.6%  16.0, 16.4%  11.3, 12.1, 17.5% |
| ([Keymer, Linhart, Rintelen, Stumpf, & Widermann, 1989](#_ENREF_6)) | Cereals  Cereals  Root crops  Root crops | Non-turning  Headland (turning)  Non-turning  Headland, turning | 5m  4m  5m  4m | 8%  20%  8%  50% |
| ([Klare, Roggendorf, Tietz, & Wollenweber, 2005](#_ENREF_7)) | Cereals  Cereals  Potato  Potato  Sugar beet  Sugar beet  Maize  Maize | Non-turning  Headland, turning  Non-turning  Headland, turning  Non-turning  Headland, turning  Non-turning  Headland | 1m  6m  1m  6m  1m  6m  1m  6m | 35%  25%  100%  83%  50%  43%  26%  30% |
| ([Raatz et al., 2019](#_ENREF_9)) | Winter wheat | Headland (turning), and non-turning | Variable | 7.5-17.5% |
| ([Sparkes, Jaggard, Ramsden, & Scott, 1998](#_ENREF_10)) | Sugar beet | Headland | 9m | 19-41% |
|  | Cereals | Headland | 9m | 3–19% |
| ([Joachim Brunotte & Sommer, 2000](#_ENREF_2)) Table 1 | Winter wheat | Headland, turning | Unspec. | 5%  29% |

Boatman, N., & Sotherton, N. (1988). Agronomic consequences and costs of managing field margins for game and wildlife conservation. *Aspects of Applied Biology, 17*, 47-56.

Brunotte, J., & Sommer, C. (2000). Gute fachliche Praxis beim Einsatz leistungsfähiger Erntetechnik–Teil 2. *LANDTECHNIK–Agricultural Engineering, 55*(1), 14-16.

Brunotte, J., Weißbach, M., Rogasik, H., Isensee, E., & Sommer, C. (2000). Zur guten fachlichen Praxis beim Einsatz moderner Zuckerrüben-Erntetechnik. *Zuckerrübe, 49*(1), 34-40.

De Snoo, G. (1994a). 13 Cost-benefits of unsprayed crop edges in winter wheat, sugar beet and potatoes. *Unsprayed field margins: implications for environment, biodiversity and agricultural practice*, 167.

De Snoo, G. (1994b). *Unsprayed field margins: implications for environment, biodiversity and agricultural practice.* (PhD). Rijksuniversiteit Leiden, Ponsen & Looijen BV, Wageningen.

Keymer, U., Linhart, C., Rintelen, P., Stumpf, M., & Widermann, R. (1989). Der Einfluß der Flurbereinigung auf die Bewirtschaftung landwirtschaftlicher Betriebe in Bayern. *Bayerisches Staatsministerium für Ernährung, Landwirtschaft und Forsten (Hrsg.): Materialien zur Flurbereinigung*(16).

Klare, K., Roggendorf, W., Tietz, A., & Wollenweber, I. (2005). *Untersuchung über Nutzen und Wirkungen der Flurbereinigung in Niedersachsen: Endbericht für ein Forschungsvorhaben im Auftrag des Niedersächsischen Ministeriums für den ländlichen Raum, Ernährung, Landwirtschaft und Verbraucherschutz*. Retrieved from

Lebert, M., Brunotte, J., & Sommer, C. (2003). *Ableitung von Kriterien zur Charakterisierung einer schädlichen Bodenveränderung, entstanden durch nutzungsbedingte Verdichtung von Böden/Regelungen zur Gefahrenabwehr: Abschlussbericht; Förderkennzeichen (UFOPLAN) 20071245*: Inst. für Betriebstechn. und Bauforschung, Bundesforschungsanst. für Landwirtschaft (FAL).

Raatz, L., Bacchi, N., Pirhofer Walzl, K., Glemnitz, M., Müller, M. E. H., Joshi, J., & Scherber, C. (2019). How much do we really lose?—Yield losses in the proximity of natural landscape elements in agricultural landscapes. *Ecology and Evolution, 9*(13), 7838-7848. doi:10.1002/ece3.5370

Sparkes, D. L., Jaggard, K. W., Ramsden, S. J., & Scott, R. K. (1998). The effect of field margins on the yield of sugar beet and cereal crops. *Annals of Applied Biology, 132*(1), 129-142. doi:DOI 10.1111/j.1744-7348.1998.tb05190.x
